# Impact of IDO activation and alterations in the kynurenine pathway on hyperserotonemia, NAD^+^ production, and AhR activation in autism spectrum disorder

**DOI:** 10.1038/s41398-023-02687-w

**Published:** 2023-12-09

**Authors:** Jean-Marie Launay, Richard Delorme, Cécile Pagan, Jacques Callebert, Marion Leboyer, Nicolas Vodovar

**Affiliations:** 1 Université Paris Cité and Inserm UMR-S 942 MASCOT, Paris, France; 2 Université Paris Cité and Child and Adolescent Psychiatry, Robert Debré Hospital, Assistance Publique-Hôpitaux de Paris, Paris, France; 3 Service de Biochimie et Biologie Moléculaire, Centre de Biologie et de Pathologie Est, Hospices Civils de Lyon, 69500 Bron, France; 4 Department of Biochemistry, Hôpital Lariboisière - Fernand Widal, Assistance Publique-Hôpitaux de Paris, Paris, France; 5 Université Paris Est Créteil and Inserm U955, IMRB, Translational Neuropsychiatry, Créteil, France; 6 AP-HP, DMU IMPACT, FHU ADAPT, Hôpitaux Universitaires Henri Mondor, Créteil, France; 7 Fondation FondaMental, Créteil, France

**Keywords:** Autism spectrum disorders, Physiology

## Abstract

Hyperserotonemia is the most replicated biochemical anomaly associated with autism spectrum disorder (ASD) and has been reported in 35–46% of individuals with ASD. Serotonin is synthesised from the essential amino acid tryptophan (TRP). However, the main catabolic route of TRP is the kynurenine pathway (KP), which competes with serotonin synthesis when indoleamine dioxygenase (IDO) is activated. Using the same cohort of individuals with ASD, we used to report extensive studies of the serotonin/melatonin pathway, and found increased kynurenine (KYN), suggesting IDO activation in 58.7% of individuals with ASD (159/271), supported by a strong negative correlation between KYN/TRP ratio and miR-153-3p plasma levels, which negatively regulates IDO. IDO activation was associated with normoserotonemia, suggesting that IDO activation could mask hyperserotonemia which meant that hyperserotonemia, if not masked by IDO activation, could be present in ~94% of individuals with ASD. We also identified several KP alterations, independent of IDO status. We observed a decrease in the activity of 3-hydroxyanthranilate dioxygenase which translated into the accumulation of the aryl hydrocarbon receptor (AhR) selective ligand cinnabarinic acid, itself strongly positively correlated with the AhR target stanniocalcin 2. We also found a deficit in NAD^+^ production, the end-product of the KP, which was strongly correlated with plasma levels of oxytocin used as a stereotypical neuropeptide, indicating that regulated neuropeptide secretion could be limiting. These results strongly suggest that individuals with ASD exhibit low-grade chronic inflammation that is mediated in most cases by chronic AhR activation that could be associated with the highly prevalent gastrointestinal disorders observed in ASD, and explained IDO activation in ~58% of the cases. Taken together, these results extend biochemical anomalies of TRP catabolism to KP and posit TRP catabolism as a possible major component of ASD pathophysiology.

## Introduction

Autism spectrum disorder (ASD) is a complex and heterogeneous neurodevelopmental disorder that is characterised by impaired social communication, restrictive interest, repetitive behaviours, and hypersensitivity [1]. The prevalence of ASD has been gradually increasing to currently reaching >1% [2]. Despite the existence of genetic components underlying ASD [3], only a few mutations are causative and responsible for 10–20% of cases at most [4]. Currently, the diagnosis of ASD is solely based on clinical evaluation by an expert physician. However, several biomarkers (oxidative metabolism, tryptophan/large neutral amino acid ratio, etc.) have been associated with autistic traits. In particular, hyperserotonemia is the most consistent biological finding [5–9] that has been reported in 30–50% of individuals with ASD [5] and has been associated with decreased sulphotransferase activities [10].

**Fig. 1:**
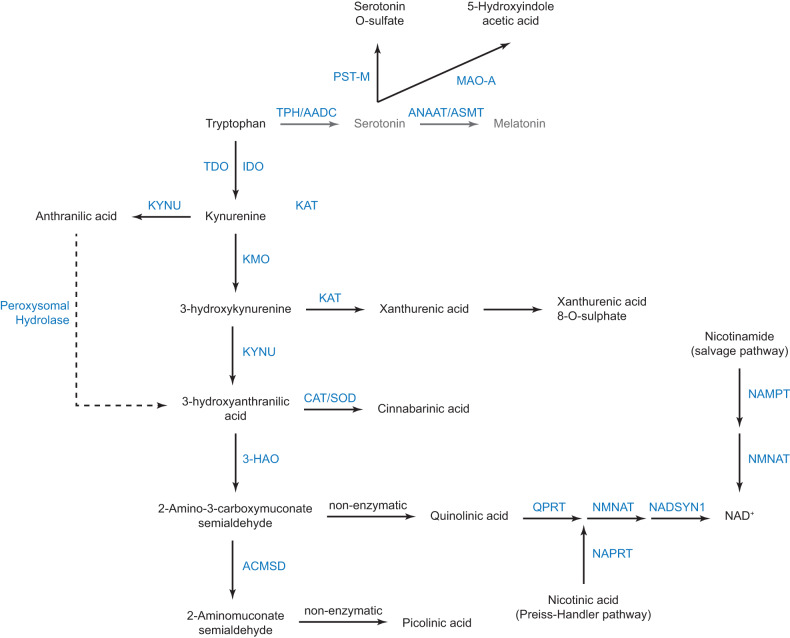
Schematic representation of the kynurenine pathway. The serotonergic pathway that also branches out of TRP is indicated in grey. Metabolites are shown in black and enzymes are in blue. 3-HAO 3-hydroxyanthranilate oxidase (EC 1.10.3.5), ACMSD aminocarboxymuconate-semialdehyde decarboxylase (EC 4.1.1.45), CAT catalase (EC 1.11.1.6), IDO indoleamine 2,3-dioxygenase (EC 1.13.11.52), KAT kynurenine-oxoglutarate transaminases or kynurenine aminotransferases (EC 2.6.1.7), KMO kynurenine 3-monooxygenase (EC 1.14.13.9), KYNU kynurenine hydrolase or kynureninase (EC 3.7.1.3), NAD^+^ nicotinamide adenine dinucleotide, MAO-A monoamine oxidase A (E.C. 1.4.3.4), NADSYN1 glutamine-dependent NAD^+^ synthetase (EC 6.3.5.1), NAMPT nicotinamide phosphoribosyl transferase (EC 2.4.2.12), NAPRT nicotinate phosphoribosyl transferase (EC 6.3.4.21), NMNAT nicotinamide nucleotide adenylyl transferase (EC 2.7.7.1), PST-M monoamine-sulphating phenol sulphotransferase (EC 2.8.2.1), QPRT quinolinate phosphoribosyl transferase (EC 2.4.2.19), SOD superoxide dismutase (EC 1.15.1.1), TDO tryptophan 3,3-dioxygenase (EC 1.13.11.11). The dotted line indicates a minor reaction.

Serotonin (5-hydroxytryptamine, 5-HT) is synthesised by tryptophan hydroxylases (TPH) and aromatic amino acid decarboxylase from the essential amino acid tryptophan (TRP), which has been established as a major regulator of innate and adaptive immunity [11]. The 5-HT pathway leads to the production of melatonin whose levels were consistently lower in ASD [5, 12]. However, the 5-HT pathway is only one of the three major metabolic pathways of TRP in humans, besides protein synthesis. The two other pathways are the transformation of TRP into several catabolites by the gut microbiota [13] or into kynurenine (KYN) by the endogenous kynurenine pathway (KP, Fig. 1) through tryptophan- and/or indoleamine-2,3-dioxygenases (TDO, IDO) [14]. Under physiological conditions, TDO converts TRP into KYN, mainly in the liver, while IDO is induced by inflammation (IFNγ, IL6) amongst other stimuli, and is also negatively regulated by miR-153-3p [15]. KYN is further metabolised by kynurenine aminotransferases (KAT), kynureninase (KYNU) and kynurenine 3-monooxygenase (KMO) to produce kynurenic acid (KA), anthranilic acid (AA), and 3-hydroxykynurenine (3-HK), respectively. 3-HK is further metabolised by KYNU into 3-hydroxyanthranilic acid (3-HAA), which serves as the substrate for the direct production of quinolinic acid (QA) and the production of picolinic acid (PA) by aminocarboxymuconate semialdehyde decarboxylase (ACMSD); production of PA by ACMSD preventing the toxic effects of QA [16]. The main output of the KMO pathway is the de novo synthesis of NAD^+^ from QA, the product of 3-HAA by 3-hydroxyanthranilate dioxygenase (3-HAO) (Fig. 1). NAD^+^ is essential for cell physiology as it is involved in multiple processes that include energy metabolism, regulation of gene expression, and secretion [17]. Importantly, many KP metabolites are ligands for the Aryl hydrocarbon Receptor (AhR), which integrates environmental cues and regulates the transcription of numerous genes involved in particular in metabolism, the inflammatory and the immune responses [18]. Quantitatively, KP is the main route of non-proteinogenic use of TRP and is influenced by both the immune system and the gut microbiota, whose dysfunctions have been implicated in the aetiology of ASD [19, 20]. There is an antagonistic effect of IDO activity on TPH activity, *i.e*. the induction of IDO leads to a decrease in TPH activity, presumably via the competition of both enzymes for TRP [21]. To date, only a few studies have investigated KP in ASD [22–29], often partially and with conflicting results. Therefore, using the same cohort of individuals with ASD in which we reported extensive studies of the 5-HT/melatonin pathway [5, 10, 12, 30], we measured KP metabolites and sought relationships with the 5-HT/melatonin pathway.

## Materials and methods

### Study population

This study was carried out according to the Declaration of the current revision of the Helsinki and approved by Local Institutional Review Boards (Comité de Protection des Personnes Ile de France IX). Written informed consent was obtained after oral and written information from all participants, their parents, or legal guardians. Clinical evaluations and blood sampling of individuals with ASD (according to DSM-IVTR criteria) and control individuals from the general population investigated for genetics and blood biochemistry have previously been detailed [5] and the population characteristics are presented in Supplementary Table 1. Venous blood samples were collected after a 48-h diet that excluded important sources of serotonin and tryptophan. Fasting blood samples were collected into tubes containing 109 mM sodium citrate (Greiner Bio-One) with a 9:1 blood-to-anticoagulant ratio. After removing 0.5 mL of whole blood (for serotonin measurements), the remainder was centrifuged at 1000 × *g* for 10 min, and the supernatant was then centrifuged at 3000 × *g* for 15 min to isolate platelets and obtain platelet-poor plasma. Importantly, the time between blood sampling and platelet/plasma isolation was less than 1 h. Whole blood, platelets, and plasmas were aliquoted into barcode-labelled cryovials and stored at −80 °C until investigations. All biomarkers measurements were performed blinded from clinical status.

### Platelet sample preparation

Platelet lysis was achieved by -SH-activated toxin treatment [31]. 150 haemolytic units of alveolysin purified to homogeneity (equivalent to about 375 ng or 6 pmol of protein) was added (10:1) to platelet suspensions at 4 °C and the suspensions were warmed to 37 °C just before measurements. This amount of toxin binds to platelets at 4 °C and elicits complete lysis at 37 °C since about 16 molecules of toxin are sufficient to lyse one human platelet [32]. Under the conditions employed here, NAD^+^ determinations (LC/MS identical to plasma) were linear with platelet protein concentrations ranging from 0.01 to 0.1 mg of protein per mL.

### TRP metabolism investigation

TRP, 5-HT, and KYN were measured by HPLC, as previously described [33]. The KYN/TRP ratio, calculated from absolute plasma concentrations of KYN and TRP, was used as an index of IDO/TDO activity, and IDO activation was considered when KYN/TRP > 5%. Values of whole-blood 5-HT [5] and platelet PST-M activities [10] were previously published. Age-dependent cut-offs for 5-HT (830 nM for <16 years old, 655 nM for >16 years old) were used to define hyperserotonemia, as previously reported [5]. The KP metabolites were quantified by LC-MS/MS as previously described [34]. For plasma 5-HIAA determinations, plasma was deproteinized by incubation with two volumes of 0.5 M HClO_4_ supplemented with 0.06 mM ascorbic acid, vortex-mixed and centrifuged at 12,000 g for 5 min. The collected supernatants were filtered through a 10-kDa membrane (Nanosep, Pall Corp., Port Washington, NY, USA) by centrifugation at 7000 g. Samples were next assessed for monoamine and metabolite content by ultra-high-performance liquid chromatography (UPLC Ultimate 3000, Thermo Scientific) and coulometric detection on a Thermo Scientific Hypersil BDS C 18 column including a Hypersil BDS guard column (analytical conditions: isocratic flow at 0.5 mL/min, oven temperature 35 °C, Thermo Scientific phase test, analytical cells potentials 100, 250, 450, and 700 mV).

### Cytokine measurements

The biomarker analyses were blinded. Plasma levels of IL-1β, IL-3, IL-4, IL-5, IL-6, IL-10, IL-12p70, IL12-p19, IL-13, IL-22, IL-33, TNFα and IFNγ were measured using sandwich immunoassay methods with commercially available electro-chemiluminescent detection systems on the Meso Scale Discovery U-PLEX platform (Meso-Scale Discovery® (MSD), Gaithersburg, USA), as per the manufacturer’s instructions. Briefly, 50 μL of plasma was loaded per well in the MSD plates. The plates were analysed using the SECTOR Imager 2400.

### Plasma NAD^+^, oxytocin and stanniocalcin-2 (STC2) measurements

NAD^+^ concentrations were determined by LC-MS as described previously [35]. Oxytocin plasma concentrations were measured by ELISA (ref: ADI-901-153A, Enzo Life Sciences, Villeurbanne, France). Solid-phase extraction of samples (600 μL) was performed using 200 mg C18 Sep-Pak Vac 3cc columns (Waters Corporation, Milford, MA, USA) and evaporated using a Speed Vac. Samples were reconstituted in 250 µL of assay buffer and assayed in triplicate per the manufacturer’s protocol. Plasma concentrations of STC2 were determined by ELISA (ref AL-143, Ansh Laboratories, Webster, TX, USA).

### miR-153-3p measurement

Total RNA was extracted from plasma samples using the mirVana PARIS kit (ref: AM1556, Ambion, Thermo Fisher Scientific, St-Quentin en Yvelines, France). The samples were spiked with synthetic *C. elegans* miRNA controls (cel-miR39, 54, and 238) at a final concentration of 2 pM to correct for extraction efficiency. After DNAse treatment, RNAs were reverse transcribed with the miScript reverse transcription kit (Qiagen, Courtaboeuf, France). cDNA was diluted 10 folds before quantitative PCR using the miScript SYBR Green PCR kit (Qiagen). A control without reverse transcriptase and a control without RNA was added to each PCR plate to ensure the absence of contaminating DNA and to check for non-specific amplification, respectively. The cycling conditions for the amplification were an initial denaturation at 95 °C for 1 min followed by 35–40 cycles [denaturation: 95 °C for 15 s, annealing: 58 °C for 60 s and extension: 72 °C for 20 s]. Expression values were normalised using the mean Ct of the spiked-in controls and calculated with the 2^−ΔCt^ formula.

### Rats

Animal care and experimental procedures complied with the European Communities Council Directive (CEE 86/609/EEC), EU Directive 2017/32/EU, and French Departmental Direction of Animal Protection (2019-11 #1027). The developmental hyperserotonemia (DHS) rat model for ASD was performed as previously described [36]. On PND21, pups were subjected to blood withdrawal to assess peripheral serotonin and other biochemical tests. Nulliparous Sprague–Dawley female rats (180–220 g) were from Charles River and housed in light- (on from 0800–2000 h) and temperature (21–24 °C)-controlled testing rooms, with food and water available *ad libitum*. They were mated overnight, and fertilisation was determined using a vaginal smear; this day was considered gestational day 0 (GD 0). Eight pregnant females received subcutaneously a single dose (1.0 mg/ kg of body weight) of 5-methoxytryptamine (5-MT, Sigma Aldrich St. Louis, MO, USA) daily from GD 12 to the day of delivery, and five pregnant females received the vehicle in equal volumes on the same days. Upon parturition (postnatal day [PND] 0), litters were culled into five males and five females. Male pups exposed to 5-MT in utero were also administered 5-MT from PND0 to PND20, to induce DHS. Similarly, pups only exposed to the vehicle prenatally were administered the vehicle in equal volumes. A single dose of interferon-gamma (12.5 µg/ kg body weight, R&D) was injected intraperitoneally at PDN21 before blood sampling. Sample size, age and sex of the animal was estimated based on previously published data [36]. Animals were not randomised.

### Statistical analysis

All statistical analyses were performed with statistical software R (version 4.2.1). Data normality was assessed using the Shapiro–Wilk test. Intergroup comparisons were performed using the rank-sum Wilcoxon test or the Kruskal–Wallis test, followed by the rank-sum Wilcoxon test corrected for multiple comparisons (Holm). Relationships between variables were evaluated using Spearman’s correlation coefficient (ρ) or linear regression (R^2^). A *p*-value < 0.05 was considered statistically significant.

## Results

### IDO activation in ASD

**Fig. 2:**
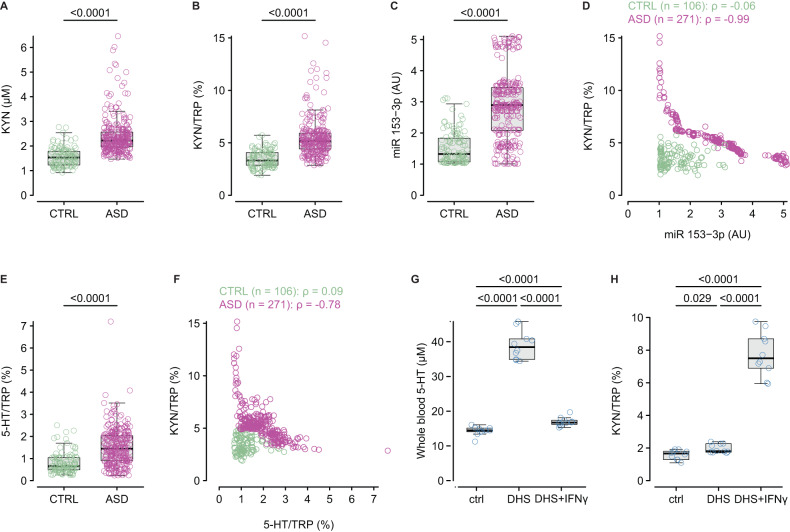
IDO activation in individuals with ASD. **A** Plasma kynurenine (KYN) levels in controls (*n* = 106) and individuals with ASD (*n* = 271). **B** IDO activity estimated by the kynurenine/ tryptophan (KYN/TRP) ratio in controls (*n* = 106) and individuals with ASD (*n* = 271). **C** Plasma miR-153-3p levels in controls (*n* = 106) and individuals with ASD (*n* = 271). **D** Relationship between the KYN/TRP ratio and plasma miR-153-3P in controls (green) and individuals with ASD (magenta). **E** TPH activity estimated by the whole blood serotonin (5-HT)/TRP ratio in controls (*n* = 106) and individuals with ASD (*n* = 271). **F** Relationship between IDO (KYN/TRP) and TPH (5-HT/TRP) activities in controls (green) and individuals with ASD (magenta). **G** Whole blood serotonin in control rats (*n* = 10), DHS rats (*n* = 10), and DHS rats that received interferon-gamma (IFNγ) (*n* = 10). **H** IDO activity (KYN/TRP) in control (*n* = 10), DHS rats (*n* = 10), and DHS rats that received IFNγ (*n* = 10). Intergroup comparisons were performed using the sum-rank Wilcoxon test (**A**–**C**, **E**) or the Kruskal–Wallis test followed by the sum-rank Wilcoxon test corrected for multiple comparisons (**G**, **H**). Correlations (**D**, **F**) were calculated using Spearman’s correlation coefficient (ρ).

KYN and TRP measurements were available for 271 individuals with ASD and 106 controls from the general population. There was no difference in TRP plasma levels between individuals with ASD and controls **(**ASD: 43.4 µM [39.8–48.9]; controls: 43.75 µM [40.8–49.0]; *P* = 0.36, Supplementary Fig. 1A), while KYN was higher in individuals with ASD than in controls (Fig. 2A). Consequently, the KYN/TRP ratio (a surrogate of TDO/IDO activity) was higher in individuals with ASD (Fig. 2B), and KYN/TRP > 5% was observed in 58.7% of individuals with ASD (*n* = 159/271, Table 1) and only in 1.9% of controls (*n* = 2/106). Importantly, TRP plasma levels were lower in individuals with ASD and KYN/TRP > 5% (Supplementary Fig. 1B). Furthermore, miR-153-3p was also higher in individuals with ASD than in controls (Fig. 2C), and strongly negatively correlated with KYN/TRP, only in individuals with ASD (Fig. 2D). Finally, cytokine profiling in a subset of individuals with ASD (*n* = 24) showed a proinflammatory profile in those with KYN/TRP > 5% (Supplementary Fig. S2), and there were strong correlations between KYN/TRP and cytokines, in particular a very strong positive correlation between KYN/TRP and IFNγ (Supplementary Fig. S3).

**Table 1:** Individuals with ASD (*n* = 271) according to their KYN/TRP and 5-HT status.

	plasma KYN/TRP ≤ 5%	plasma KYN/TRP > 5%	Total
Whole-blood 5-HT ≤ 16 y/o: ≤830 nM > 16 y/o: ≤655 nM	16 (5.9%)	142 (52.4%)	158 (58.3%)
Whole-blood 5-HT ≤ 16 y/o: >830 nM > 16 y/o: >655 nM	96 (35.4%)	17 (6.3%)	113 (41.7%)
Total	112 (41.3%)	159 (58.7%)	271 (100%)

TPH activity, estimated by the 5-HT/TRP ratio, was higher in individuals with ASD when compared to controls (Fig. 2E). The KYN/TRP and 5-HT/TRP ratios were inversely correlated, only in individuals with ASD (Fig. 2F). When considering hyperserotonemia (based on age-dependent cut-offs^5^) and KYN/TRP, 94% of individuals with ASD (*n* = 255/271) had hyperserotonemia and/or KYN/TRP > 5%, of which 87.8% had either KYN/TRP > 5% and normoserotonemia or KYN/TRP ≤ 5% and hyperserotonemia (Table 1). To test the potential role of MAO-A in hyperserotonemia, we measured 5-Hydroxyindoleacetic acid (5-HIAA), the MAO-A product of 5-HT, in the plasma and found that it was higher in individuals with ASD than in controls (Supplementary Fig. 4A). To confirm the impact of IDO activation on serotonemia in ASD, we used the developmental hyperserotonemia (DHS) rat model of ASD, which exhibits hyperserotonemia (Fig. 2G). DHS rats had similar plasma TRP levels (Supplementary Fig. 4B) and moderately higher plasma KYN levels (Supplementary Fig. 4C) compared to control rats, resulting in a moderate increase in the KYN/TRP ratio in DHS rats compared to controls (Fig. 2H). However, both KYN (<4 µM in rats) and KYN/TRP remained within the normal range, indicating that IDO was not activated in the DHS model. When IDO was pharmacologically activated in DHS rats by a single intraperitoneal injection of interferon-gamma (12.5 µg/kg of body weight, Fig. 2H and Supplementary Fig. 2B, C), whole blood serotonin markedly decreased and returned to values only moderately higher than controls (Fig. 2G).

### KP main metabolites

**Fig. 3:**
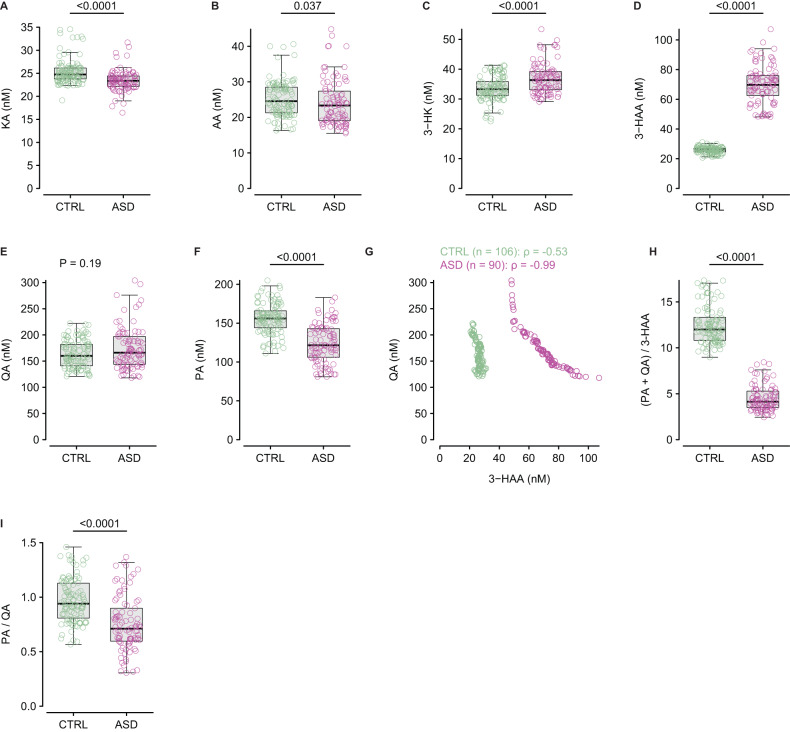
KP main metabolites. Plasma levels of kynurenic acid (KA, **A**), anthranilic acid (AA, **B**), 3-hydroxykynurenine (3-HK, **C**), 3-hydroxyanthranilic acid (3-HAA, **D**), quinolinic acid (QA, **E**), and picolinic acid (PA, **F**) in controls (*n* = 106) and individuals with ASD (*n* = 90). **G** Relationship between plasma QA and 3-HAA in controls (*n* = 106) and individuals with ASD (*n* = 90). **H** 3-HAO activity estimated by the (PA + QA)/3-HAA ratio in controls (*n* = 106) and individuals with ASD (*n* = 90). **I** ACMSD activity estimated by the PA/QA ratio in controls (*n* = 106) and individuals with ASD (*n* = 90). Intergroup comparisons were performed using the sum-rank Wilcoxon test. The correlation was calculated using Spearman’s correlation coefficient (ρ).

KP measurements were available in 90 individuals with ASD and 106 controls due to sample exhaustion. In this subset of individuals with ASD, TRP was similar compared to controls (Supplementary Fig. 5A), while KYN (Supplementary Fig. 5B) and KYN/TRP ratio (Supplementary Fig. 5C) were higher than in controls, and 63% of individuals with ASD (*n* = 57/90) had KYN/TRP > 5%. These data were similar to those of the entire cohort (Supplementary Fig. 1A and Fig. 2A, B). Regarding the main KP metabolites, plasma KA (Fig. 3A) and AA (Fig. 3B) levels were lower in individuals with ASD than in controls. In contrast, the first two metabolites of the KMO pathway, 3-HK (Fig. 3C) and 3-HAA (Fig. 3D), were higher in individuals with ASD than in controls. Notably, there was no overlap in 3-HAA plasma concentration between individuals with ASD and controls. Concerning the terminal metabolites of the KMO pathway, QA was similar between individuals with ASD and controls (Fig. 3E), while PA was lower in individuals with ASD when compared to controls (Fig. 3F), which markedly contrasted with the lack of 3-HAA overlap between the two groups. The difference in 3-HAA and PA levels in individuals with ASD and controls, and the similar QA levels observed strongly suggest that 3-HAO and ACMSD activities are decreased in ASD. Indeed, there was a strong negative correlation between 3-HAA and QA, only in ASD (Fig. 3G), and the (PA + QA)/3-HAA, an estimator of 3-HAO activity, was markedly lower in individuals with ASD compared to controls (Fig. 3H). Similarly, ACMSD activity, estimated by the PA/QA ratio, was also lower in ASD than in controls (Fig. 3I). When considering individuals with ASD with KYN/TRP ≤ 5% and >5%, there was no difference for any of the main KP metabolites between both groups (Supplementary Fig. 6). When considering the panel of cytokine tested, there were no correlations between KA, AA, 3-HK, 3-HAA, QA, and PA and cytokines (Supplementary Fig. 3).

### NAD^+^ production is altered in ASD

**Fig. 4:**
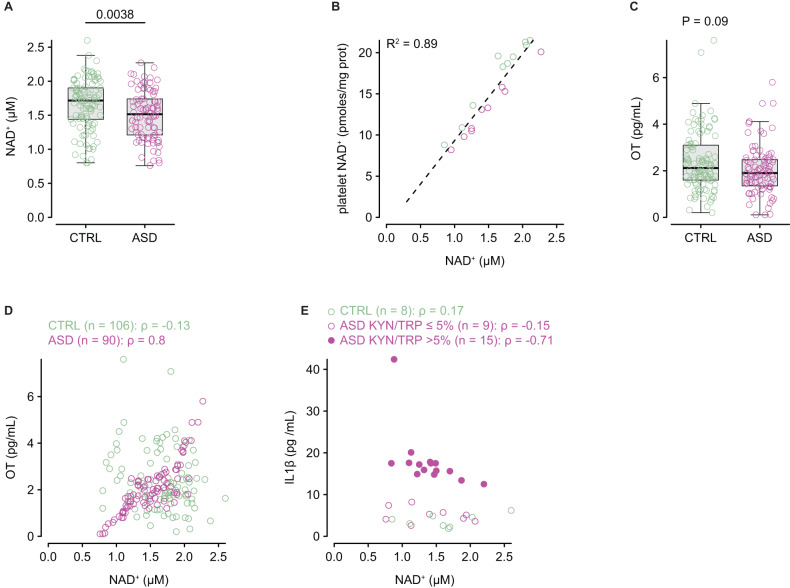
NAD^+^ production and KP minor metabolites. **A** Plasma levels of NAD^+^ in controls (*n* = 106) and individuals with ASD (*n* = 90). **B** Relationship between plasma and platelet NAD^+^ in controls (*n* = 10) and individuals with ASD (*n* = 9). **C** Oxytocin (OT) plasma levels in controls (*n* = 106) and individuals with ASD (*n* = 90). **D** Relationship between plasma NAD^+^ and OT in controls (green) and individuals with ASD (magenta). **E** Relationship between plasma NAD^+^ and IL1β levels in controls (*n* = 8), individuals with ASD and KYN/TRP ≤ 5% (*n* = 9), and individuals with ASD and KYN/TRP ≤ 5% (*n* = 15). Intergroup comparisons were performed using the sum-rank Wilcoxon test; the correlations were calculated using linear regression (R^2^) or Spearman’s correlation coefficient (ρ).

A major output of the KP is the production of NAD^+^ from QA, via the de novo NAD^+^ synthetic pathway (Fig. 1). Plasma NAD^+^ was ~11% lower in individuals with ASD compared to controls (Fig. 4A), despite similar QA plasma levels (Fig. 3E). Of note, platelet NAD^+^ very strongly positively correlated with plasma NAD^+^, both in controls and ASD (Fig. 4B). In addition to its role in energy metabolism, NAD^+^ also plays an important role in calcium homoeostasis and regulated secretion. We thus measured oxytocin as a stereotypical secreted neuropeptide and found no difference in plasma levels between individuals with ASD and controls (Fig. 4C). However, we observed a very strong positive relationship between NAD^+^ and oxytocin, only in individuals with ASD (Fig. 4D). Finally, there was a strong negative correlation between NAD^+^ and IL1β, only in individuals with ASD and KYN/TRP > 5% (Fig. 4E).

### KP minor metabolites

**Fig. 5:**
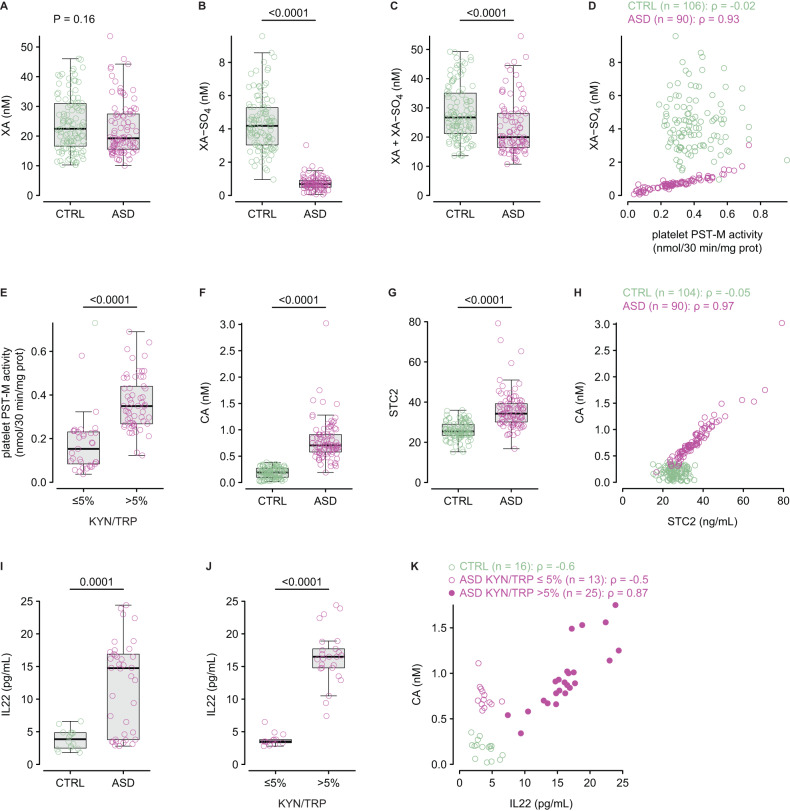
KP minor metabolites. Plasma levels of xanthurenic acid (XA, **A**), its sulphoconjugate XA-SO_4_ (**B**), and the sum of XA and XA-SO_4_ (**C**) in controls (*n* = 106) and individuals with ASD (*n* = 90). **D** Relationship between XA-SO_4_ and platelet PST-M activity in controls (green) and individuals with ASD (magenta). **E** Platelet PST-M activity in individuals with ASD with KYN/TRP ≤ 5% (*n* = 33) and > 5% (*n* = 57). **F** Plasma levels of cinnabarinic acid (CA) in controls (*n* = 106) and individuals with ASD (*n* = 90). **G** Plasma levels of STC2 in controls (*n* = 106) and individuals with ASD (*n* = 90). **H** Relationship between plasma CA and STC2 in controls (*n* = 104) and individuals with ASD (*n* = 90). **I** IL22 plasma levels in controls (*n* = 16) and individuals with ASD (*n* = 38). **J** IL22 plasma levels in individuals with ASD and KYN/TRP ≤ 5% (*n* = 13) or >5% (*n* = 25). **K** relationship between CA and IL22 in controls (*n* = 16), individual with ASD and KYN/TRP ≤ 5% (*n* = 13), and individuals with ASD and KYN/TRP > 5% (*n* = 25). Intergroup comparisons were performed using the sum-rank Wilcoxon test. Correlations were calculated using Spearman’s correlation coefficient (ρ).

Finally, we measured xanthurenic and cinnabarinic acids (XA, CA), which branch out from the main KMO pathway with 3-HK and 3-HAA as their precursors, respectively (Fig. 1). XA plasma levels were similar between individuals with ASD and controls (Fig. 5A). However, its sulphoconjugate XA-SO_4_ was lower in individuals with ASD (Fig. 5B), indicating a deficit in XA sulphoconjugation, and the sum of XA and XA-SO_4_ (Fig. 5C) was also lower in individuals with ASD, suggesting a deficit in XA production. Furthermore, there was a very strong positive relationship between plasma XA-SO_4_ levels and platelet PST-M activities, only in individuals with ASD (Fig. 5D). Importantly, XA plasma levels were unchanged when considering individuals with ASD with KYN/TRP ≤ 5% and >5% (Supplementary Fig. 7A), while both XA-SO_4_ plasma levels (Supplementary Fig. 7B) and platelet PST-M activity (Fig. 5E) were higher in individuals with ASD when KYN/TRP > 5%. The increase in platelet PST-M activity was specific as platelet PST-P activity was similar in individuals with ASD with KYN/TRP ≤ 5% and >5% (Supplementary Fig. 7C). CA was higher in individuals with ASD than in controls (Fig. 5F), and 97% (*n* = 87/90) had CA values above the 95^th^ percentile of controls. CA is a selective AhR ligand that regulates the transcription of stanniocalcin 2 (STC2) [37]. As for CA, STC2 plasma levels were higher in individuals with ASD when compared to controls (Fig. 5G), and there was a strong positive correlation between CA and STC2, only in individuals with ASD (Fig. 5H). There were no differences in CA and STC2 levels in individuals with ASD with KYN/TRP ≤ 5% and >5% (Supplementary Fig. 7D, E). IL22, which is also regulated by AhR was higher in individuals with ASD compared to controls (Fig. 5I), and amongst individuals with ASD, was higher when KYN/TRP > 5% (Fig. 5J). Furthermore, there was a very strong positive correlation between CA and IL22, only in individuals with ASD and KYN/TRP > 5% (Fig. 5K).

### Clinical correlates

We next addressed the potential relationship between IDO activity and whole blood 5-HT, and clinical phenotypes of individuals with ASD, although not all individuals with ASD were evaluated using the same scales (Supplementary Table 2). Individuals with ASD with KYN/TRP > 5% had higher self-injury subdomain scores with the Repetitive Behaviour Scale-Revised, higher verbal IQ (estimated with the age-adapted Weschler scales for verbal individuals or the PPVT and Raven’s Progressive Matrices for non-verbal-individuals), and higher non-verbal communication subdomain score with the Autism Diagnostic Interview-Revised (ADI-R). Although the number of individuals who were subjected to the subdomain scores of social responsiveness scales – 2^nd^ edition (SRS-2) scores is limited, individuals with ASD and KYN/TRP > 5% were more severe for all parameters except for the social motivation subdomain, which was similar in both groups. There was no correlation between KP metabolites and clinical scores (Supplementary Table 3), nor there was between KP metabolites, cytokines and gastrointestinal disorders as a composite of constipation, abdominal pain or diarrhoea (Supplementary Table 4).

## Discussion

In the present study, our results suggest that the KP is altered at various levels in ASD and that individuals with ASD likely exhibit low-grade chronic inflammation that is accompanied by IDO and AhR activation.

Our data strongly suggest that ~59% of the individuals with ASD have IDO activation based on KYN/TRP > 5%. This conclusion is supported by: *i)* a decrease in plasma TRP that could be indicative of excessive TRP use to fuel the KP, *ii)* a cytokine profile highly suggestive of low-grade inflammation, in particular an unfavourable IL6/IL4 ratio, and *iii)* a strong correlation between KYN/TRP and miR-153-3p, which downregulate IDO1 expression. From a clinical standpoint, IDO activation was associated with more severe forms of ASD, especially using the Social Responsiveness Scale algorithm. If confirmed, these results also indicate that hyperserotonemia itself is only a marker of ASD and may not contribute much to the pathophysiology of ASD. Interestingly, miR-153-3p has also been reported to target the leptin receptor and alleviate some autistic symptoms in the mouse valproate model of autism [38]. MiR-153-3p is, along with miR-451 [30], the second miRNA that we found dysregulated in our cohort of ASD, reassessing a possible role of miRNAs in ASD pathophysiology [39]. Importantly, the KYN/TRP ratio across 5% could almost fully dichotomise individuals with normo- and hyperserotonemia, which highly suggests that IDO activation could mask hyperserotonemia. This hypothesis is supported by: ***i***) the strong negative correlation between KYN/TRP and 5-HT/TRP ratios, reflecting the possible competition of IDO and TPH for TRP [21], and ***ii***) the almost normalisation of hyperserotonemia in the DHS rat model after experimental IDO activation by IFN-γ. These results strongly suggest that for individuals with ASD with normoserotonemia and IDO activation, the underlying cause of hyperserotonemia was originally present, but hyperserotonemia itself is no longer observed due to a secondary IDO activation. These results indicate that the underlying mechanism leading to hyperserotonemia could be much more prevalent than previously reported – although masked when IDO is activated, and may suggest greater biochemical homogeneity in ASD than initially thought. From a mechanistic standpoint, IDO activation has been associated with decreased serotonin synthesis, presumably due to competition between the two pathways for tryptophan [21]. Our data also suggest that IDO activation is associated with a specific increase in PST-M activity, which likely increases peripheral serotonin elimination, hence normoserotonemia. However, the mechanism that dictates the relationship between IDO and PST-M activity is unknown. Of note, although we cannot exclude a role of MAO-A in this context, our results suggest that MAO-A has a minor role, if any, in this context.

We also found that KP metabolites exhibit an unexpected profile in ASD, which seems to be irrespective of IDO activation, as there was no difference in metabolite levels whether IDO is induced or not. First, we found a possible decrease in KAT activity, which is illustrated by lower KA and total XA levels in ASD. However, while AA is modestly reduced in ASD, there is no clear evidence for a marked decrease in KYNU activity, since 3-HAA was higher in ASD when compared to controls. Second and most notably, 3-HAA accumulated in ASD while QA levels were similar to controls, which strongly suggests a deficit in 3-HAO activity. This possible deficit in 3-HAO is also illustrated by the strong negative relationship between 3-HAA and QA. Although the mechanism underlying those enzymatic deficits is unknown, they echoed the coregulation of KAT and 3-HAO observed in the PBMC of individuals with ASD [40], albeit both also correlated with MAO-A which does not appear to be reduced here. Additionally, the decrease in PA also suggests a deficit in ACMSD, and a cryptic structural variant in a regulatory region of *ACMSD* was associated with ASD [41]. Such a deficit in ACMSD activity likely leads to an increased QA toxicity as the decrease in PA suggests a decrease in neuroprotection to toxic QA [16]. Finally, we observed a decrease in plasma NAD^+^ levels which was previously reported from the metabolic profiling of lymphoblastoid cells [22]. The similar QA levels in controls and ASD strongly suggest that NAD^+^ production is impaired in ASD, independently of the alterations observed in the KP. However, while a deficit in QPRT could explain this decrease in NAD^+^, we cannot exclude alterations in the two other pathways as they all share same enzymes (Fig. 1). Overall, these data indicate that all branches of the TRP pathways are impaired in ASD with direct and indirect impacts on pathophysiology.

The reduction in plasma NAD^+^, which reflects a reduction in platelet and likely intracellular NAD^+^, translated into dose-dependent oxytocin plasma levels. This is also illustrated by the negative relationship between plasma NAD^+^ and IL1β, which secretion is inhibited by NAD^+^ [42]. These results suggest that the decrease in NAD^+^ production causes a decrease in the regulated secretion of numerous peptides and hormones and could be involved in the pathophysiology of ASD. In addition to energetic metabolism, NAD^+^ is also involved in numerous other biological processes and such lower levels of NAD^+^ may extend beyond regulated secretion (oxytocin and IL1 β). For instance, NAD^+^ is a cofactor for histone deacetylases [17], and NAD^+^ concentration and the NAD^+^/NADH balance are important for histone acetylation, indirectly leading to KYN-dependent epigenetic activation [43]. Furthermore, the importance of nutrient metabolites such as acetyl coenzyme A (acetyl-CoA) and S-adenosylmethionine, which are acetyl and methyl donors for post-translational modifications (acetylation, methylation), is well known in epigenetic regulation. For instance, acetyl-CoA-specific histone H4 lysine 5 acetylation and histone H3 lysine 79 methylation at the AhR-bound *stc2* promoter was recently reported [44], and we observed an increase in STC2 plasma concentration for individuals with ASD. Furthermore, KYN and 3-HK - the two KP metabolites for which a reliable concordance between peripheral and central measures was reported [45], and AA function as epigenetic modulators, increasing histone H3 lysine 4 trimethylation and histone H2A serine 40 *O*-glycosylation, resulting in up-regulated gene expression at most hypothalamic-linked loci [43]. These three KP metabolites and the resulting epigenetic modifications thus appear important for stability in gene expression. Overall, these data suggest that the KP alterations observed in our cohort are likely to translate into modifications in epigenetically regulated genes in ASD [46].

Increased CA plasma and STC2 levels are indicative of AhR activation. While AhR is an important regulator of the inflammatory and immune responses, there was a strong positive correlation between CA and the AhR transcriptional target IL22 [47], only in individuals with ASD who had KYN/TRP > 5%. This is reminiscent of platelet PST-M activity that markedly increased in individuals with ASD and KYN/TRP > 5%. Interestingly, the AhR ligand β-Naphthoflavone induced PST-M (*SULT1A3*) but not PST-P (*SULT1A1*) transcription in Hep G2 cells [48], which suggests that AhR activation by KP metabolites other than CA could be responsible for normal platelet PST-M activity, hence normal 5-HT blood levels, in individuals with ASD and IDO activation. Importantly, IL22 is involved in epithelial homoeostasis, in particular intestinal permeability, and is up-regulated in inflammatory bowel disease (IBD) [49]. Unlike other mental illnesses, ASD is associated with a high incidence of IBD [50]. Therefore, one could anticipate that individuals from the cohort with IDO/AhR activation would present with more gastrointestinal disorders. However, in this cohort, only constipation, diarrhoea and abdominal pain were recorded as a composite feature and showed no association with IDO activation or IL22 plasma levels measured in a small subset of the population. Hence, further studies assessing the association between IDO/AhR/IL22 and IBD are warranted. Nevertheless, the gut microbiome drives immunoregulation and faulty immunoregulation, as well as inflammation, predispose to psychiatric disorders, including ASD [51], while psychological stress drives further inflammation through pathways that involve the gut microbiome [52]. The chronic activation of an IDO/AhR/IL22 cascade observed in ~59% of the individuals from the present cohort may reflect an abnormal interaction with the gut microbiota. Taken together, these data suggest two modalities of AhR activation: **i**) an IDO-independent CA-dependent AhR activation in most individuals with ASD which is associated with an AhR-limited transcriptional program (e.g., STC2) whose pathophysiological consequences remain to be established, and **ii**) a broader AhR response that includes an increase in IL22 and normal PST-M levels, which is only achieved when IDO is induced. The latter likely reflects a low-grade chronic inflammatory response, which has so far been supported by little evidence [27, 53]. While constant AhR activation impacts gut physiology [54], IDO activation was associated with more severe ASD phenotypes (Supplementary Table 2). Based on these results, one may hypothesise that IDO and AhR inhibitors, which are currently being tested in cancer [55, 56], could benefit individuals with ASD although, their side effects may restrict their use [56]. This also suggests that the KYN/TRP could be biomarkers for evaluating and quantifying low-grade inflammation in ASD. However, standardised pre-analytical and analytical aspects must be considered when measuring KYN and TRP to ensure their precision and generalisation and avoid conflicting results[23, 24, 27–29].

In conclusion, the present study extends our previous findings on 5-HT and melatonin [10, 30] to show that IDO activation occurs in a majority of individuals with ASD, and is likely associated with low-grade inflammatory response. IDO activation is also associated with normal blood serotonin, possibly via the transcriptional activation of PST-M by AhR. Finally, our results show that several enzymes of the KP appear to be less functional, as previously observed for enzymes involved in the serotonin arm of TRP metabolism. It remains to establish the pathophysiological consequences of those enzymatic anomalies.

### Supplementary information

Supplementary Data

## Data Availability

Data are available upon reasonable request
