# Supplementary material for: Impact of IDO activation and alterations in the kynurenine pathway on hyperserotonemia, NAD+ production, and AhR activation in autism spectrum disorder
Source: Transl Psychiatry. 2023 Dec 9;13:380. doi: 10.1038/s41398-023-02687-w (PMC10710433; doi:10.1038/s41398-023-02687-w)
Supplement: Supplementary file 1 — Supplementary Data [file 41398_2023_2687_MOESM1_ESM.pdf]

Impact of IDO activation and alterations in the kynurenine pathway on hyperserotonemia, NAD^+ production, and AhR activation in autistic spectrum disorder

Jean-Marie Launay PhD, Richard Delorme MD PhD, Cécile Pagan PhD, Jacques Callebert PhD, Marion Leboyer MD PhD, and Nicolas Vodovar, PhD

Supplementary Table 1-4

Supplementary Fig. 1-7

Supplementary Table 1: Characteristics of the control and ASD population.

Variable	Controls	ASD	P
Age	32.7 [11.8-40.3] (n = 106)	16.1 [9.6-23.1] (n = 272)	<0.0001
Sex (M)	50 (47.2%; n = 106)	205 (81.7%; n = 251)	<0.0001
Gastrointestinal Disorders	4 (3.8%; n = 106)	16 (18.2%; n = 88)	0.0023
**Autism Diagnostic Interview – Revised version**			
Communication	N/A	16 [7-23] (n = 212)	N/A
Communication non-verbal only	N/A	8 [2-13] (n = 211)	N/A
Restricted, repetitive, and stereotyped patterns of behaviour	N/A	5 [2-7.25] (n = 212)	N/A
Abnormality of development before 36 months	N/A	4 [2-5] (n = 213)	N/A
**Social Responsiveness Scale – 2nd edition***			
Total	N/A	71.5 [64-80] (n = 50)	N/A
Social awareness	N/A	65 [54-73] (n = 55)	N/A
Social cognition	N/A	67 [58-77.5] (n = 51)	N/A
Social communication	N/A	70.5 [64-77.75] (n = 50)	N/A
Social motivation	N/A	71 [62-78] (n = 55)	N/A
Restricted, repetitive behaviours and interest	N/A	75 [63-83] (n = 53)	N/A
Social Communication and Interaction	N/A	70.5 [62.5-80] (n = 50)	N/A
**Repetitive Behaviour Scale – Revision version**			
Total score	N/A	22 [8.25-32.75] (n = 54)	N/A
Stereotypic behaviours	N/A	3.5 [1-7] (n = 66)	N/A
Self-injurious behaviours	N/A	0 [0-2] (n = 61)	N/A
Compulsive behaviours	N/A	2 [0-5] (n = 65)	N/A
Ritual behaviours	N/A	3 [1-5.75] (n = 66)	N/A
Sameness behaviours	N/A	4 [2-9.25] (n = 64)	N/A
Restricted Interests	N/A	3 [0.25-6] (n = 66)	N/A
**Intellectual Quotient (IQ)#**			
Verbal	N/A	91 [66-112] (n = 112)	N/A
Non verbal	N/A	76 [66-102] (n = 166)	N/A
Full IQ	N/A	73 [50-103] (n = 141)	N/A

Gastrointestinal disorders were recorded as a composite categorical variable matching constipation, abdominal pain or diarrhoea with no further detail. All data are raw mean score [minimal – maximal] / (number of individuals) except for * (t-score) and # (score were normalized); For IQ: we estimated IQ using Weschler scales adapted to age for verbal individuals or the Peabody Picture Vocabulary Test™ Fourth Edition & the Raven's Progressive Matrices for non-verbal individuals.

Supplementary Table 2: Comparisons of cognitive and clinical features between the hyperserotonaemia – KYN/TRP ≤ 5% and normoserotonaemia – KYN/TRP > 5% groups of individuals with ASD.

**Variable**	**High 5-HT KYN/TRP ≤ 5% (n = 96)**	**Normal 5-HT KYN/TRP > 5% (n = 182)**	**P**
**Autism Diagnostic Interview – Revised version**			
Social Interactions	20 [12 - 25.5] (n = 83)	19 [3 - 26] (n = 107)	0.76
Communication	14 [7 - 19] (n = 58)	12 [0 - 17] (n = 86)	0.26
Non-verbal communication only	10 [3.5 - 13] (n = 83)	7.5 [0 - 12] (n = 106)	**0.03**
Restricted, repetitive, and stereotyped patterns of behaviour	6 [3 - 8] (n = 83)	5 [0 - 7] (n = 107)	0.26
Abnormality of development before 36 months	4 [2 - 5] (n = 83)	3 [1.75 - 4.25] (n = 108)	0.10
**Social Responsiveness Scale – 2nd edition***			
Total	64 [59.5 - 70.25] (n = 18)	76 [69 - 83] (n = 29)	**0.004**
Social awareness	58 [52 - 66] (n = 19)	70 [61 - 78] (n = 33)	**0.009**
Social cognition	58 [53.5 - 68.5] (n = 18)	72 [65.25 - 81.75] (n = 30)	**0.007**
Social communication	66 [58.75 - 71] (n = 18)	71 [65 - 80] (n = 29)	**0.03**
Social motivation	69 [59.5 - 77] (n = 19)	75 [64 - 80] (n = 33)	0.26
Restricted, repetitive behaviours and interest	63.5 [60.5 - 74.25] (n = 18)	78 [73 - 83.5] (n = 32)	**0.003**
Social Communication and Interaction	64.5 [57.8 - 71.8] (n = 18)	73 [66 - 83] (n = 29)	**0.01**
**Repetitive Behaviour Scale – Revision version**			
Total	11 [7.5 - 27] (n = 16)	25.5 [15.5 - 34] (n = 34)	0.09
Stereotypic behaviours	2 [1 - 5] (n = 20)	4 [1 - 7] (n = 41)	0.17
Self-injurious behaviours	0 [0 - 0] (n = 19)	1 [0 - 2.75] (n = 38)	**0.01**
Compulsive behaviours	2 [0 - 6] (n = 19)	3 [0 - 5] (n = 41)	0.95
Ritual behaviours	2.5 [0.75 - 3.5] (n = 20)	4 [1 - 6] (n = 41)	0.16
Sameness behaviours	3 [1.25 - 7.75] (n = 18)	4 [2 - 10] (n = 41)	0.26
Restricted Interests	2 [0 - 5.5] (n = 19)	3.5 [1.25 - 6] (n = 42)	0.39
**Intellectual Quotient (IQ)#**			
Verbal IQ	79.5 [49.8 - 98.8] (n = 36)	96 [76.3 - 112] (n = 58)	**0.007**
Non-verbal	66 [65 - 94] (n = 57)	76 [66 - 101.5] (n = 87)	0.11
Full IQ	65 [41 - 98] (n = 51)	76.5 [49.75 - 104] (n = 68)	0.15

All data are raw mean score [minimal – maximal] / (number of individuals) except for * (t-score) and # (score were normalized); For IQ: we estimated IQ using Weschler scales adapted to age for verbal individuals or the Peabody Picture Vocabulary Test™ Fourth Edition & the Raven’s Progressive Matrices for non-verbal individuals.

Supplementary Table 3: Correlation (Spearman) between cognitive and clinical features, and KP metabolites in individuals with ASD.

Score	PA	QA	KA	AA	3-HK	3-HAA	XA	XA-SO_4	CA	NAD	OT	STC2
**Autism Diagnostic Interview – Revised version**												
Social Interaction	-0.12	0.08	-0.03	-0.15	0.05	-0.08	0.07	-0.14	0.09	-0.06	-0.05	0.12
Communication	-0.1	0.02	-0.16	-0.07	0.05	-0.03	0.12	-0.11	0.17	-0.07	-0.11	0.22
Communication non-verbal only	-0.09	0.04	-0.1	-0.04	0.05	-0.04	0.18	-0.12	0.14	-0.05	-0.11	0.18
Restricted, repetitive, and stereotyped patterns of behaviour	-0.16	0.03	-0.15	-0.06	0.04	-0.03	0.18	-0.14	0.16	0	-0.06	0.19
Abnormality of development before 36 months	-0.47	0.2	0.01	0.01	-0.1	-0.2	0.01	-0.25	0.18	0.04	0.12	0.23
**Social Responsiveness Scale – 2nd Edition***												
Total Score	-0.34	0.19	-0.01	-0.27	-0.06	-0.2	-0.32	0.1	0.09	0.38	0.49	0.08
Social awareness	-0.32	0.08	0.21	-0.36	-0.14	-0.1	-0.15	0.17	-0.09	0.14	0.23	-0.13
Social cognition	-0.3	0.21	0.11	-0.15	-0.21	-0.23	-0.3	0.29	0.11	0.35	0.4	0.09
Social communication	-0.26	0.14	0.05	-0.36	0.03	-0.14	-0.31	0.06	0.09	0.35	0.46	0.12
Social motivation	-0.01	-0.04	-0.02	-0.08	0.24	0.06	-0.42	-0.06	-0.12	0.11	0.16	-0.09
Restricted, repetitive behaviours and interest	-0.43	0.13	-0.14	-0.12	-0.14	-0.14	-0.34	0.01	0.27	0.21	0.35	0.26
Social Communication and Interaction	-0.28	0.16	0.02	-0.3	0	-0.17	-0.3	0.08	0.02	0.37	0.44	0.02
**Repetitive Behaviour Scale – Revision version**												
Total Score	-0.16	0.03	-0.06	0.1	-0.04	-0.06	-0.16	0.11	-0.07	0.16	0.27	-0.06
Stereotypic behaviours	-0.32	-0.02	0.19	0.03	-0.04	0.02	-0.11	-0.08	-0.22	0.18	0.11	-0.2
Self-injurious behaviours	-0.29	-0.03	-0.1	0.07	0.02	0.01	-0.15	0.2	-0.09	0.18	0.3	-0.06
Compulsive behaviours	-0.03	-0.06	-0.01	0.26	-0.01	0.04	-0.02	-0.02	-0.26	-0.02	0.02	-0.27
Ritual behaviours	-0.21	0.06	-0.07	0.08	0.05	-0.09	-0.2	-0.07	0.14	0.24	0.23	0.14
Sameness behaviours	-0.07	-0.02	-0.07	0.07	0.01	-0.01	-0.23	0.02	0.06	0.05	0.19	0.07
Restricted Interests	-0.3	0.03	-0.02	0.15	0.01	-0.04	-0.25	-0.02	-0.01	0.12	0.16	0.01
**Intellectual Quotient (IQ) #**												
Verbal	0.27	-0.04	0.26	0.05	-0.06	0.04	0.29	0.42	-0.04	-0.13	-0.12	-0.05

Non-verbal	0.13	-0.09	0.13	-0.06	0.03	0.1	0.14	0.16	-0.16	-0.12	-0.09	-0.17
Full IQ	0.29	-0.16	0.2	-0.02	0.07	0.16	0.23	0.31	-0.11	-0.18	-0.17	-0.11

All data are raw mean score [minimal – maximal] / (number of individuals) except for * (t-score) and # (score were normalized); For IQ: we estimated IQ using Weschler scales adapted to age for verbal individuals or the Peabody Picture Vocabulary Test™ Fourth Edition & the Raven's Progressive Matrices for non-verbal individuals. PA: picolinic acid, QA: quinolinic acid, AA: anthranilic acid, 3-HK: 3-hydroxykynurenine, 3-HAA: 3-hydroxyanthranilic acid, XA: xanthurenic acid, XA-SO_4: xanthurenic acid sulphate, CA: cinnabarinic acid, NAD: Nicotinamide adenine dinucleotide, OT: oxytocin, and STC2: stanniocalcin 2.

Supplementary Table 4: Comparisons of KP and cytokines between individuals with ASD with or without gastrointestinal disorders (composite variable matching constipation, abdominal pain or diarrhoea with no further detail). Variables are expressed as median [interquartile range]. The number of individuals involved in each comparison is indicated.

Variables	No GI disorders	GI disorders	P
Tryptophan (µM)	42.1 [39.6-47.25] (n = 72)	41.2 [38.8-46.675] (n = 16)	0.52
Kynurenine (µM)	2.285 [2.0275-2.66] (n = 72)	2.385 [2.135-2.575] (n = 16)	0.78
IDO	5.4 [4.8-6.1] (n = 72)	5.3 [4.8-6.4] (n = 16)	0.81
KA (nM)	22.9 [21.955-24.375] (n = 55)	23.5 [23.31-23.9675] (n = 12)	0.45
AA (nM)	23.47 [19.25-28] (n = 55)	24.075 [18.925-25.75] (n = 12)	0.85
3-HK (nM)	35.4 [32.25-39.15] (n = 55)	36.45 [31.525-37.55] (n = 12)	0.76
3-HAA (nM)	66 [57.35-74.35] (n = 55)	71.45 [61.725-75.4] (n = 12)	0.6
QA (nM)	183 [151-203.5] (n = 55)	162.5 [142.75-197.5] (n = 12)	0.57
PA (nM)	121 [112-139.5] (n = 55)	132 [109.5-144.25] (n = 12)	0.65
XA (nM)	22 [16.9-29.4] (n = 55)	18.2 [15.2-23] (n = 12)	0.11
XA-S04 (nM)	0.69 [0.47-0.895] (n = 55)	0.635 [0.4875-0.735] (n = 12)	0.52
CA (nM)	0.76 [0.605-0.92] (n = 55)	0.695 [0.6425-0.9375] (n = 12)	0.64
NAD (µM)	1.46 [1.205-1.795] (n = 55)	1.6 [1.56-1.9125] (n = 12)	0.13
OT (pg/mL)	1.81 [1.355-2.58] (n = 55)	1.94 [1.725-2.5025] (n = 12)	0.55
STC2	36.3 [31.55-40.05] (n = 55)	33.55 [31.3-38.775] (n = 12)	0.52
IL-22	15.2 [4.2-17.35] (n = 31)	6.5 [4.2-13.85] (n = 7)	0.15
IL1b (pg /mL)	15.25 [6.875-17.5] (n = 20)	9.1 [5.175-13.3] (n = 4)	0.23
IL3 (pg /mL)	2.1 [1.575-2.8625] (n = 20)	1.95 [1.8-3.3] (n = 4)	0.73
IL4 (pg /mL)	3.45 [1.775-4.65] (n = 20)	5.35 [3-7.75] (n = 4)	0.28
IL5 (pg /mL)	49.1 [25.75-58.575] (n = 20)	48.6 [42.95-55.25] (n = 4)	0.85
IL6 (pg /mL)	9.9 [3.43-16.55] (n = 20)	9.45 [5.595-13.525] (n = 4)	0.74
IL10 (pg /mL)	3.55 [1.8-9.675] (n = 20)	6.2 [1.175-12.325] (n = 4)	0.91
IL12 (p70) (pg /mL)	23.45 [16.2-29] (n = 20)	20.75 [16.8-24.225] (n = 4)	0.46
IL13 (pg /mL)	5.1 [3.24-9.975] (n = 20)	9.45 [5.15-16.6] (n = 4)	0.31
IL12 (p19) (pg /mL)	300 [229.25-320.9] (n = 20)	247.3 [217.825-260.25] (n = 4)	0.21
IL33 (pg /mL)	12.6 [8.075-16.2] (n = 20)	10.4 [8.375-13.375] (n = 4)	0.73
TNFα (pg/mL)	24.45 [17.25-32.4] (n = 20)	25.4 [17.975-33.225] (n = 4)	0.97
IFNγ (pg/mL)	21.95 [16.175-25.975] (n = 20)	22.75 [16.2-28.8] (n = 4)	0.85

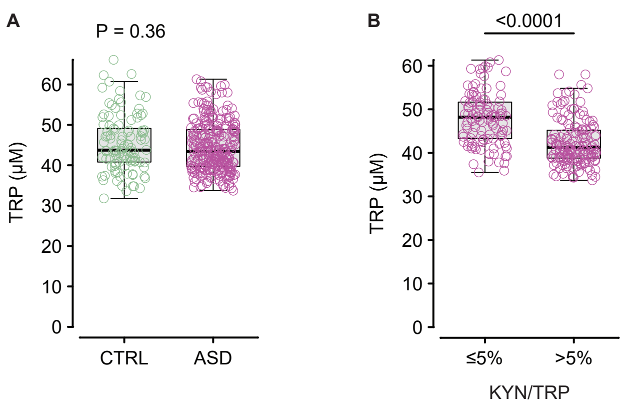

Supplementary Fig. 1: A, Tryptophan (TRP) plasma levels in controls (n = 106) and individuals with ASD (n = 271). **B,** TRP plasma levels in individuals with ASD and KYN/TRP ratio ≤ 5% (n = 112) and > 5% (n = 159). Comparisons were performed using the sum-rank Wilcoxon test.

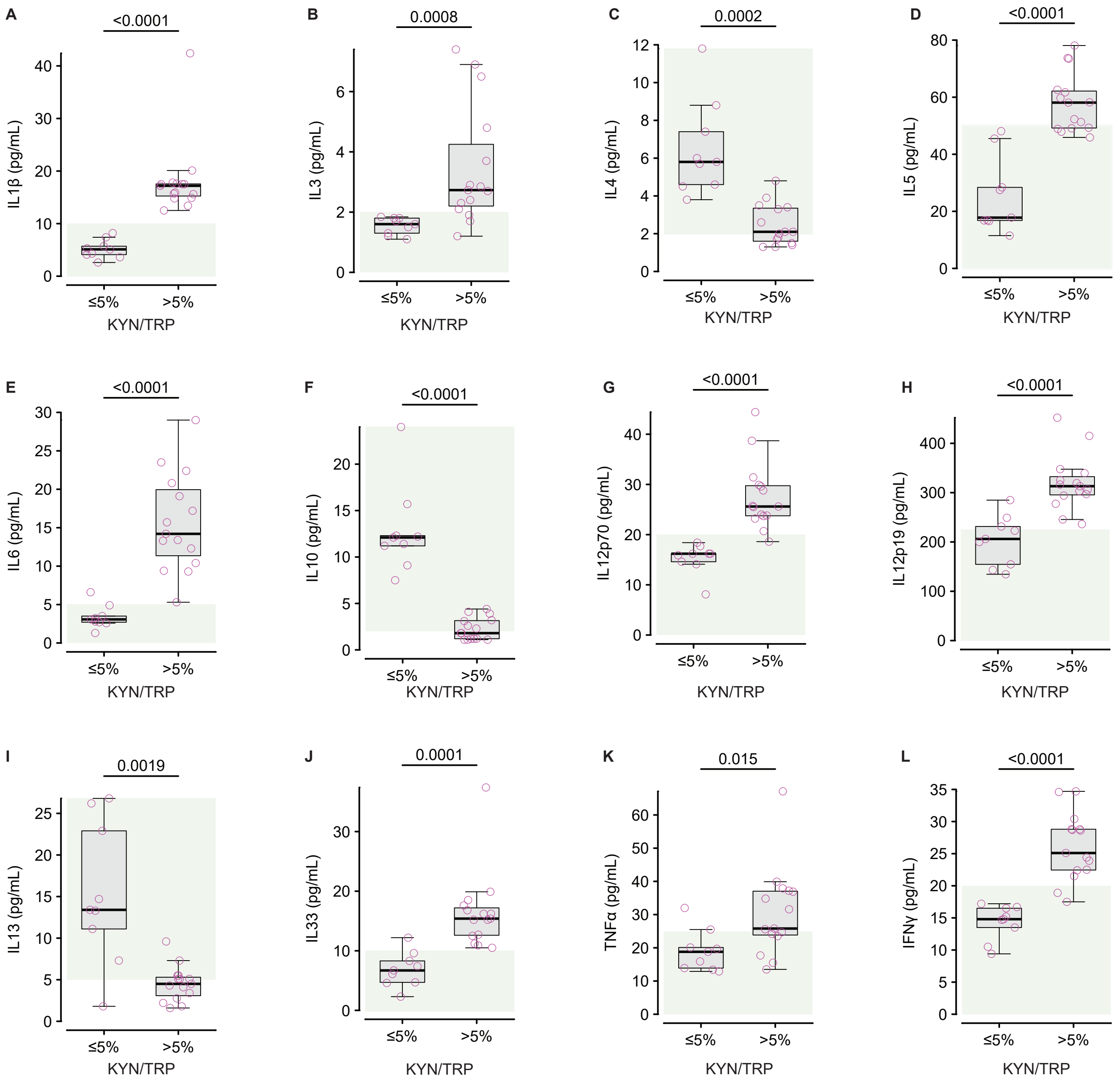

Supplementary Fig. 2: A-L, plasma levels of cytokines in a subset of individual with ASD and KYN/TRP ratio ≤ 5% (n = 9) and > 5% (n = 15). Comparisons were performed using the sum-rank Wilcoxon test. The reference range for the cytokines is indicated as a shaded rectangle.

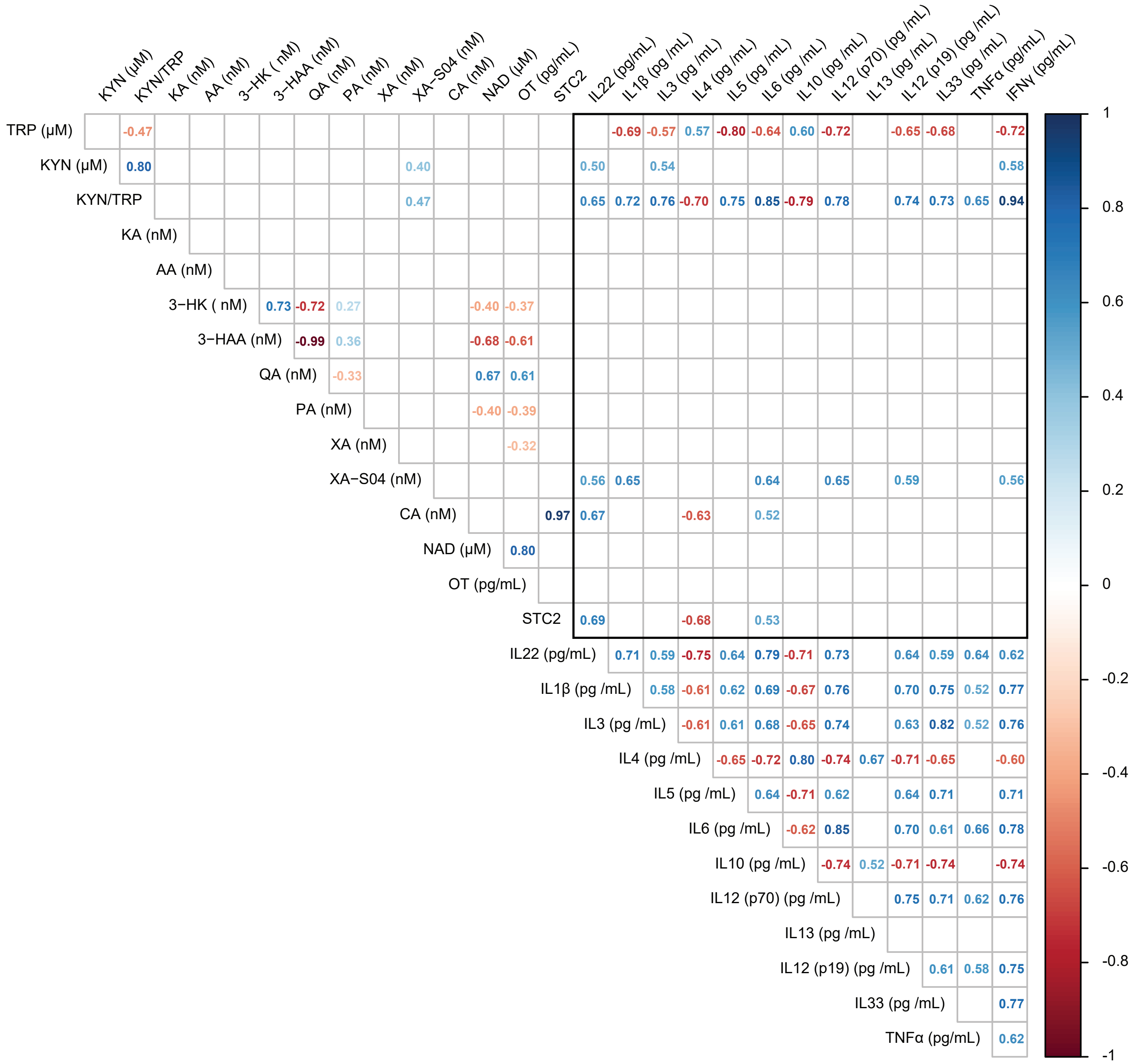

Supplementary Fig. 3: Correlation matrix that includes KP metabolites and cytokines in individuals with ASD. Correlations were calculated using the Spearman's correlation coefficient. Only correlation with P < 0.01 are indicated. TRP, KYN, and TRP/KYN were measured in 271 individuals with ASD; other KP metabolites, NAD^{+}, OT, and STC2 were measured in 90 indididuals with ASD; cytokines, except IL22, were measured in 24 indididuals with ASD; IL22 was measured in 38 individuals with ASD. The number of individuals per correlation is the lowest in which each of the two parameters were measured. The black square highlights the correlations between KP metabolites and cytokines (n = 24).

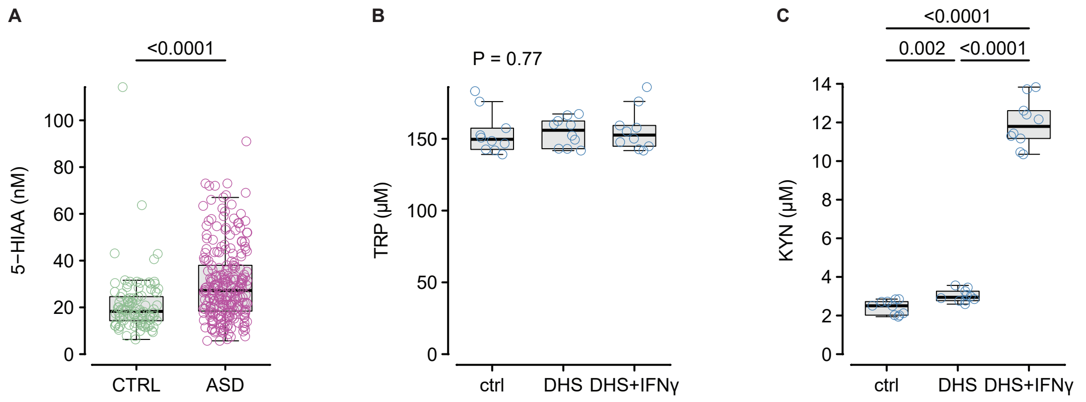

Supplementary Fig. 4: A, 5-hydroxyindole acetic acid plasma levels in controls (n = 98) and individuals with ASD (n = 237). **B,** TRP plasma levels in control rats (n = 10), DHS rats (n = 10), and DHS rats that received interferon gamma (IFNγ) (n = 10). **C,** Plasma levels of kynurenine (KYN) in control rats (n = 10), DHS rats (n = 10), and DHS rats that received IFNγ (n = 10). Comparisons were performed using the sum-rank Wilcoxon test (a) or the Kruskal-Wallis test followed by the sum rank Wilcoxon test corrected for multiple comparisons (b-c).

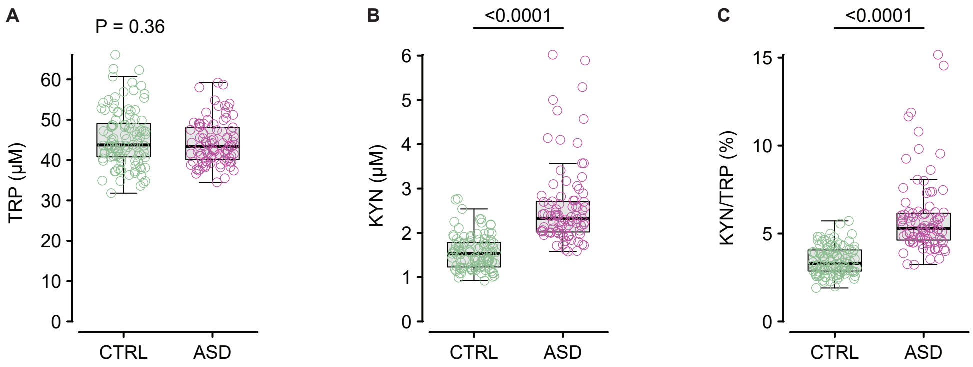

Supplementary Fig. 5: Plasma levels of tryptophan (TRP, **A**), kynurenine (KYN, **B**), and KYN/TRP ratio as a surrogate for IDO activity (**C**) in controls (n = 106) and individuals with ASD for whom KP measurements were available (n = 90). Comparisons were performed using the sum rank Wilcoxon test.

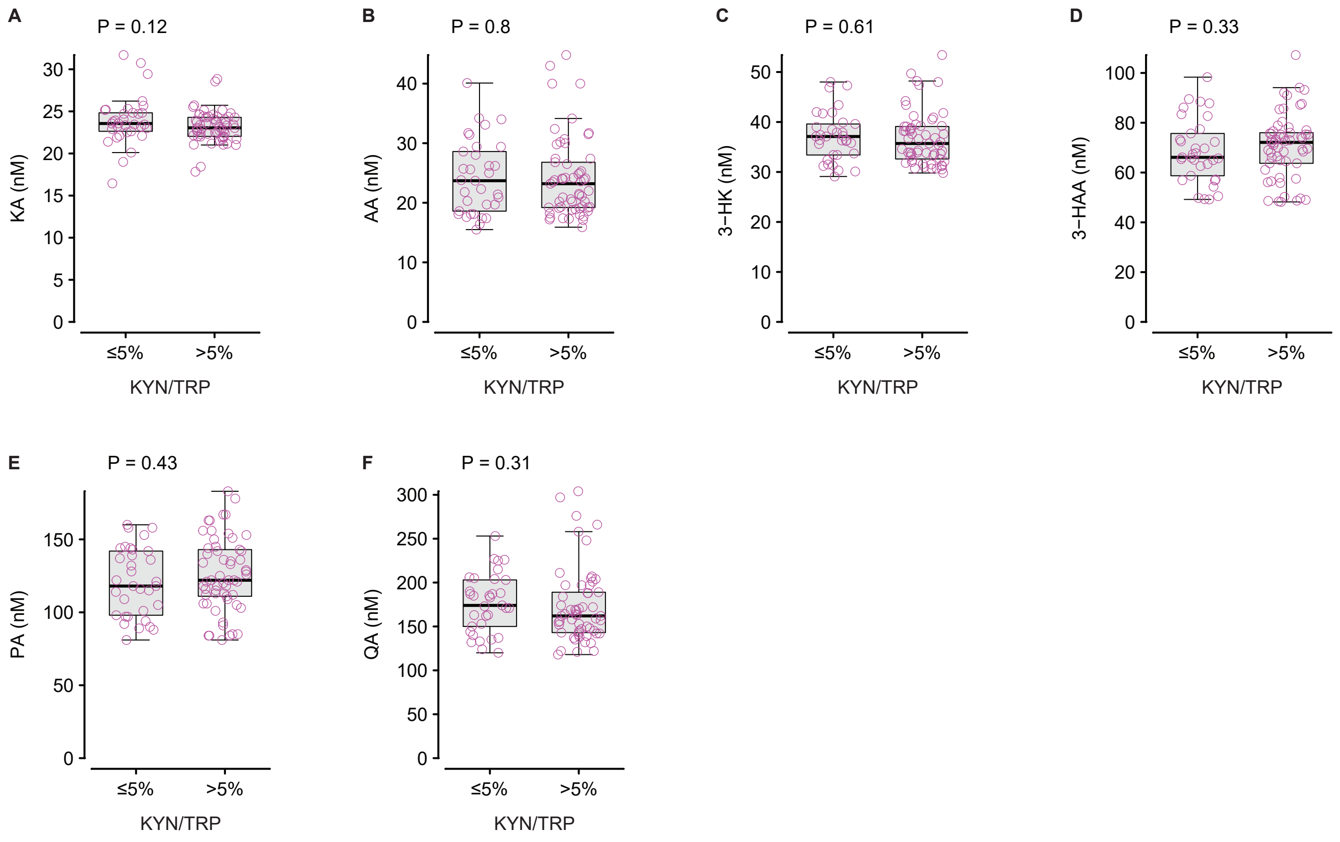

Supplementary Fig. 6: Comparison of plasma kynurenic acid (KA, **A**), anthranilic acid (AA, **B**), 3-hydroxykynurenine (3-HK, **C**), 3-hydroxyanthranilic acid (3-HAA, **D**), picolinic acid (PA, **E**), and quinolinic acid (QA, **F**) in individuals with ASD with KYN/TRP ratio ≤ 5% (n = 33) and > 5% (n = 57). Intergroup comparisons were performed using the sum-rank Wilcoxon test.

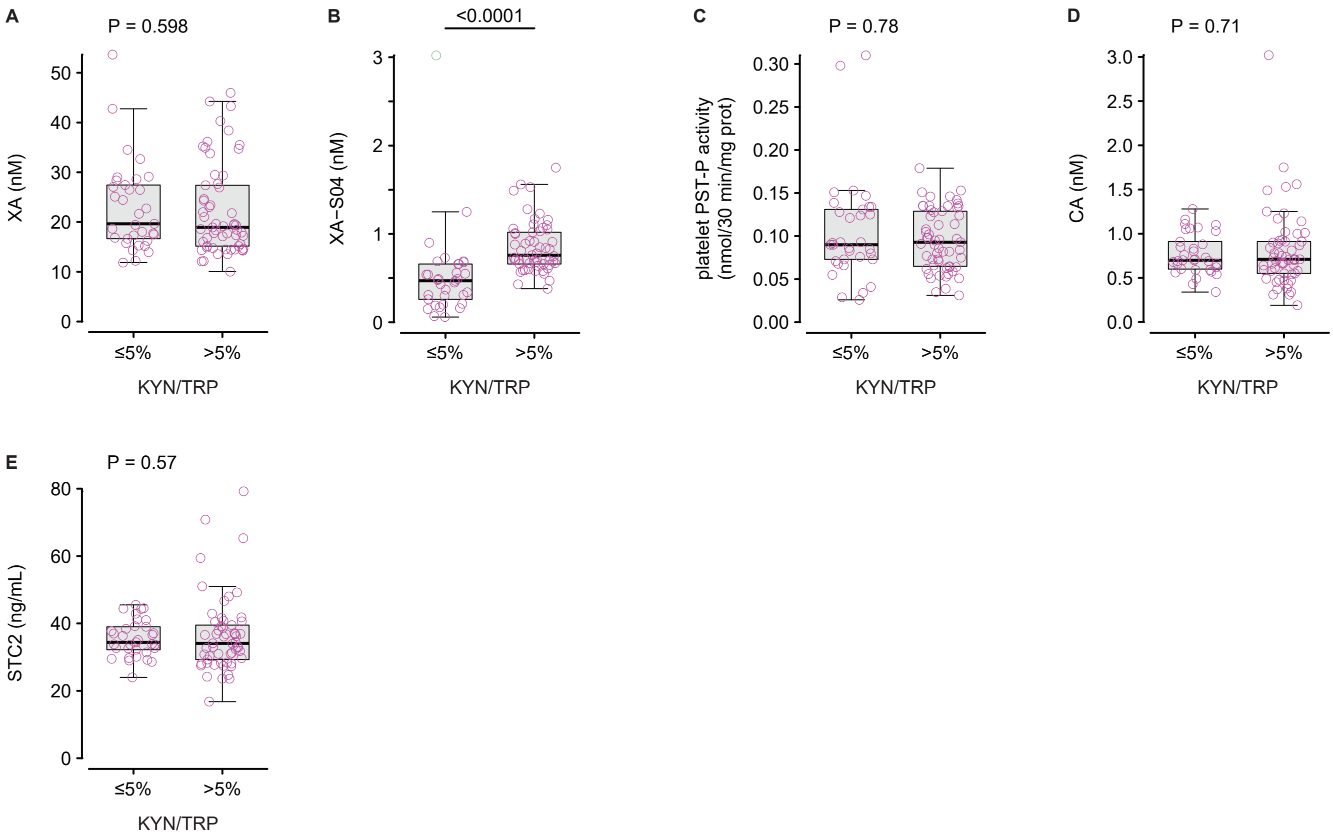

Supplementary Fig. 7: A, Plasma levels of xanthurenic acid (XA) in individuals with ASD with KYN/TRP ratio ≤ 5% (n = 33) and > 5% (n = 57). **B,** Plasma level of XA-SO_4 in individuals with ASD with KYN/TRP ≤ 5% (n = 33) and > 5% (n = 57). **C,** Platelet PST-P activity in individuals with ASD with KYN/TRP ratio ≤ 5% (n = 33) and > 5% (n = 57). **D,** Plasma levels of cinnabarinic acid (CA) in individuals with ASD with KYN/TRP ratio ≤ 5% (n = 33) and > 5% (n = 57). **E,** Plasma STC2 levels in individuals with ASD with KYN/TRP ratio ≤ 5% (n = 33) and > 5% (n = 57). Intergroup comparisons were performed using the sum-rank Wilcoxon test.
